# ADAM9 Up-Regulates N-Cadherin via miR-218 Suppression in Lung Adenocarcinoma Cells

**DOI:** 10.1371/journal.pone.0094065

**Published:** 2014-04-04

**Authors:** Yuh-Pyng Sher, Li-Ju Wang, Li-Ling Chuang, Mong-Hsun Tsai, Ting-Ting Kuo, Cheng-Chung Huang, Eric Y. Chuang, Liang-Chuan Lai

**Affiliations:** 1 Graduate Institute of Clinical Medical Science, China Medical University, Taichung, Taiwan; 2 Center for Molecular Medicine, China Medical University Hospital, Taichung, Taiwan; 3 Graduate Institute of Physiology, National Taiwan University, Taipei, Taiwan; 4 Department of Physical Therapy and Graduate Institute of Rehabilitation Science, Chang Gung University, Taoyuan, Taiwan; 5 Institute of Biotechnology, National Taiwan University, Taipei, Taiwan; 6 Graduate Institute of Biomedical Electronics and Bioinformatics, National Taiwan University, Taipei, Taiwan; 7 Bioinformatics and Biostatistics Core, Center of Genomic Medicine, National Taiwan University, Taipei, Taiwan; H.Lee Moffitt Cancer Center & Research Institute, United States of America

## Abstract

Lung cancer is the leading cause of cancer death worldwide, and brain metastasis is a major cause of morbidity and mortality in lung cancer. CDH2 (N-cadherin, a mesenchymal marker of the epithelial-mesenchymal transition) and ADAM9 (a type I transmembrane protein) are related to lung cancer brain metastasis; however, it is unclear how they interact to mediate this metastasis. Because microRNAs regulate many biological functions and disease processes (e.g., cancer) by down-regulating their target genes, microRNA microarrays were used to identify ADAM9-regulated miRNAs that target CDH2 in aggressive lung cancer cells. Luciferase assays and western blot analysis showed that *CDH2* is a target gene of miR-218. MiR-218 was generated from pri-mir-218-1, which is located in *SLIT2*, in non-invasive lung adenocarcinoma cells, whereas its expression was inhibited in aggressive lung adenocarcinoma. The down-regulation of *ADAM9* up-regulated *SLIT2* and miR-218, thus down-regulating *CDH2* expression. This study revealed that *ADAM9* activates *CDH2* through the release of miR-218 inhibition on *CDH2* in lung adenocarcinoma.

## Introduction

Lung cancer represents the leading cause of cancer-related death in the Western world. This disease has a 5-year overall survival rate of only 15%, and this has not improved during recent decades [1]. In Taiwan, lung cancer is also the leading cause of cancer death [2], and adenocarcinoma is the major histological type (52.5%). Metastasis is a major cause of morbidity and mortality in lung cancer. Surgical resection of primary lung cancer is frequently followed by tumor recurrence at distant sites, such as the lymph nodes [3], bone [4], and brain [5]. Approximately 30% of patients with lung cancer develop brain metastasis [5]. However, the mechanisms mediating lung cancer metastasis to the brain remain unclear.

Cancer invasion into distant sites requires the degradation of extracellular matrix components, which may be mediated by matrix metalloproteinases, and the loosening of epithelial cell-cell junctions and adhesions to generate mesenchymal cell types, which is referred to as the epithelial-mesenchymal transition [6], [7]. Currently, several genes related to lung cancer brain metastases have been identified, such as *CDH2* and *ADAM9* [8], [9]. Neural cadherin (N-cadherin), encoded by the *CDH2* gene, is a transmembrane protein and plays an important role in cell adhesion [10]. In most cancers, the expression of *CDH2* increases during tumor progression [11] and induces cell migration and invasion as a mesenchymal marker in the epithelial-mesenchymal transition [6], [12]. These observations indicate that CDH2 plays a critical role in metastasis [11], [12]; therefore, its expression needs to be tightly regulated. *CDH2* expression can be regulated by methylation, transcription factors, and microRNAs (miRNAs). For example, the expression of *CDH2* in gastric cancer cells was up-regulated following demethylation [13]. Additionally, *CDH2* expression is regulated by several transcription factors, such as Twist 1 [14], TP63 [15], and CTNNB1 [16]. Currently, little is known about how miRNAs regulate *CDH2*. Only miR-145 has been reported to target *CDH2* in gastric cancer [17], and it remains unclear whether other microRNAs can regulate *CDH2*.

MiRNAs are a class of small non-coding RNAs that are approximately 22 nucleotides in length [18] and originate from longer primary miRNA transcripts located in either intergenic or intronic regions. Intergenic miRNAs are located in the regions between genes, and intronic miRNAs are found in the introns of genes [19]. Intronic miRNAs are co-expressed with the genes in which they are located and are regulated from the same promoters as their host genes [19]. Initially, the primary miRNA is transcribed in the nucleus, is modified by the RNAase III endonuclease Drosha, and subsequently forms a hairpin-like precursor miRNA (pre-miRNA) [20]. Pre-miRNAs are transported to the cytoplasm by exportin 5, where they are further modified into their mature form by dicer. The mature miRNA combines with the RNA-induced silencing complex (RISC) and suppresses its target mRNAs by binding the 3' untranslated region (3′-UTR) of the target genes. This binding leads to the suppression of translation and/or the degradation of the mRNA [21].

A disintegrin and metalloprotease 9 (ADAM9) is a member of the ADAM family of type I transmembrane proteins and plays an important role in the regulation of the cell–cell and cell–matrix interactions that are critical determinants of malignancy. The disintegrin domain of ADAM9 adheres to cells by binding to integrins [22], and the metalloprotease domain functions by releasing a variety of cell surface proteins, such as growth factors, cytokines, cell adhesion molecules, and receptors [23]. Overexpression of ADAM9 has been observed in many cancers [24] and is correlated with brain metastasis [8]. However, the molecular mechanism underlying this association is not clearly understood.

In the current study, we aimed to better understand the relationship between CDH2 and ADAM9 in lung cancer brain metastasis. We hypothesized that miRNAs may play a role in ADAM9-CDH2 regulation, and we identified several differentially expressed miRNAs in aggressive lung adenocarcinoma using miRNA microarrays. We further demonstrated that ADAM9 could inhibit the expression of miR-218 and its precursor pri-miR-218-1 and could, in turn, up-regulate the expression of *CDH2* to increase the mobility of lung adenocarcinoma cells.

## Materials and Methods

### Cell culture

Several human lung adenocarcinoma cell lines were used, including A549, H1299, CL1-0, F4, and BM7. A549 and H1299 cells were obtained from Bioresource Collection and Research Center (Hsinchu, Taiwan). BM7 cell line was a brain-metastatic clone derived from a high metastatic subline F4, which had higher invasion capability than its parental cell line CL1-0. CL1-0 cells were a gift from Dr. Pan-Chyr Yang (National Taiwan University, Taipei, Taiwan) [25]. F4 cells with stable high level luciferase expression were established as previously described [26].

The human lung cancer cell lines CL1-0, A549, and H1299 were maintained in RPMI-1640 medium (GIBCO, Carlsbad, CA, USA) supplemented with 10% fetal bovine serum (FBS) and 1% antibiotics (GIBCO, Carlsbad, CA, USA) at 37°C in a humidified incubator under 5% CO_2_. The brain metastatic lung adenocarcinoma cell line BM7 and its parental cell line F4 were cultured in complete DMEM/F12 media (GIBCO) containing 10% FBS and 1% antibiotics (penicillin-streptomycin solution, Biological Industries, Beit-Haemek, Israel). All cell lines were authenticated by short tandem repeat (STR) DNA typing (Genelabs Life science, Taipei, Taiwan) in November 2013.

### Illumina human v2 microRNA expression beadchip and data analysis

Cells were flash frozen in liquid N_2_ and stored at −80°C until RNA extraction. Total RNA was extracted using TRIZOL Reagent (Ambion, Carlsbad, CA, USA). The RNA concentration and quality were determined using a NanoDrop ND-1000 spectrophotometer (NanoDrop Technologies, Wilmington, DE) and an Agilent 2100 Bioanalyzer (Agilent Technologies, Palo Alto, CA), which was used to calculate an RNA integrity number (RIN). Total RNA with an A260/A280 between 1.7 and 2.1 and a RIN >7.0 was adjusted to 40–200 ng/μl with DEPC-treated H_2_O. A total of 1 μg of RNA was used for the microRNA assay. Input RNA was polyadenylated and converted into cDNA using standard methods. A single miRNA-specific oligo (MSO) was used to assay each miRNA on the panel. All MSOs were hybridized to the sample in parallel, and a solid-phase primer extension step further increased the specificity and reduced the noise. After eluting the extended products and performing PCR with fluorescently labeled universal primers, the double-stranded PCR products were bound to a solid phase, and the labeled, single-stranded PCR products were prepared for Human v2 microRNA expression beadchip hybridization (Illumina, San Diego, CA). After 14–20 hours of hybridization, the beadchip was washed and coated with xylene solution. The intensities of the bead fluorescence were determined using the Illumina BeadArray Reader, and the results were analyzed using GenomeStudio v2010.1 software. The microarray data in this study are MIAME compliant [27] and have been submitted to the Gene Expression Omnibus (GEO) database (accession number GSE51666).

Quantile normalization was performed using Partek Genomics software (Partek, St. Louis, MO, USA). MiRNAs were selected when their expression change was greater than 2-fold in the three miRNA microarrays. The array results from the brain metastatic lung adenocarcinoma cells were compared to the results from the parental F4 cell line.

### Quantitative reverse transcription PCR

Total RNA was extracted using TRIZOL Reagent (Ambion, Carlsbad, CA, USA) according to the manufacturer's instructions. Reverse transcription of total RNA and microRNA was performed using the High Capacity cDNA RT Kit (Applied Biosystems, Foster City, CA, USA) and the TaqMan MicroRNA Reverse Transcription kit (Applied Biosystems, Foster City, CA), respectively. The resulting cDNA was detected using the FastStart Universal SYBR Green Master Mix (Roche, Branchburg, NJ, USA) or Universal ProbeLibrary Probe #21 (Roche, Branchburg, NJ, USA) with a 7900 Fast Real-Time PCR system (Applied Biosystems, Foster City, CA). MiR-191 and 18S rRNA were used as endogenous controls to normalize the expression of miRNA and mRNA, respectively. The following primers were used for miRNA detection: miR-218: 5′-GCGGCTTTGTGCTTGATCTAA-3′ (forward), 5′-GTGCAGGGTCCGAGGT-3′ (reverse); Pri-mir-218-1: 5′-GTGATAATGTAGCGAGATTTCTG-3′ (forward), 5′-TGTAGAAAGCTGCGTGAC-3′ (reverse); and Pri-mir-218-2: 5′-GACCAGTCGCTGCGGGGCT-3′ (forward), 5′-TGCAGGAGAGCACGGTGCTTTCCG-3′ (reverse). The following primers were used for mRNA detection: *CDH2*, set I: 5′-CCATCAAGCCTGTGGGAATC-3′ (forward), 5′-GCAGATCGGACCGGATACTG-3′ (reverse) [28]; set II: 5′-CTCCATGTGCCGGATAGC-3′ (forward), 5′-CGATTTCACCAGAAGCCTCTAC-3′ (reverse); *SLIT2*: 5′-GAACATAACACTTCAGATTGCCAC-3′ (forward), 5′-CACCATCCACGGACAAAGAG-3′ (reverse); *SLIT3*: 5′-GCTCATCACTGTCAACTTCGT-3′ (forward), 5′-CTGTCTCCACACTGTACACTG-3′ (reverse); and *ADAM9*: 5′-CCCCCAAATTGTGAGACTAAAG-3′ (forward), 5′-TCCGTCCCTCAATGCAGTAT-3′ (reverse).

### Construct design and cell transfection

#### MiR-218-expressing vector

The primary sequence of miR-218, including the flanking precursor sequence (110 bp long, MI0000295), was amplified from human leukocyte DNA. The following primers with *BamH*I and *Bgl*II restriction sites were used: 5′-TTCTGAGGATCCGTGGAGGCACCTTTTCCATA-3′ (forward) and 5′- ATTCTAAGATCTTTCACAGCTAGTCACACAATGG-3′ (reverse). The 600-bp PCR product was gel-purified and cloned into the *BamH*I-*Bgl*II sites of the pcDNA6.2-GW/EmGFP-miR-neg vector (Invitrogen, Carlsbad, CA, USA). The tetracycline-induced miR-218 plasmid (pAS4.1w.Ppuro-aOn-pri-miR218) was constructed by inserting the pri-miR218 PCR fragment into the pAS4.1w.Ppuro-aOn vector through NheI and EcoRV digestion. The pAS4.1w.Ppuro-aOn plasmid was obtained from the National RNAi Core Facility (Academia Sinica, Taiwan). The lentiviral tet-on-miR218 plasmid was used to infect BM7 cells to generate stable cell lines.

#### Luc-*CDH2* vector

The *CDH2* 3′-UTR was amplified by PCR from genomic DNA isolated from human blood. The pMIR-*CDH2*-3′UTR construct was digested with *Spe*I and *Mlu*I, and the generated fragment was inserted into the *Spe*I-*Mlu*I sites of the pMIR-REPORT miRNA Expression Reporter Vector (Applied Biosystems, Carlsbad, CA, USA). Three miR-218 binding sites in the *CDH2* 3′-UTR were predicted using miRSystem [29], and these sites were located at 2,671–2,691 bp, 2,740–2,760 bp, and 3,571–3,591 bp relative to the transcription start site. Mutations were made in the miR-218 binding sites in the *CDH2* 3′-UTR using the QuikChange Site-Directed Mutagenesis Kit (Agilent Technologies, Santa Clara, CA, USA) according to the manufacturer's protocol.

#### Cell transfection

BM7 and H1299 cells were seeded in antibiotic-free medium at 70–80% confluence. The cells were transfected with using Lipofectamine LTX with Plus Reagent (Invitrogen, Carlsbad, CA, USA) according to the manufacturer's instructions.

#### MiR-218 mimic and inhibitor transfection

Lung cancer cells were grown in antibiotic-free medium at 70–80% confluence in 6-well plates. Indicated cells were transfected with miR-218 mimic (Ambion), miR-218 inhibitor (Ambion), or negative control (Ambion) using Lipofectamine 2000 transfection reagent (Invitrogen, Carlsbad, CA, USA) according to the manufacturer's instructions.

### shRNA-mediated gene silencing of *ADAM9*

HEK293T packaging cells (ATCC # CRL-11268) were cultured in high-glucose DMEM supplemented with 10% FBS. HEK293T were transfected using Turbofect (Thermo Scientific) according to the manufacturer's instructions. The specific lentiviral shRNA constructs targeted against *ADAM9* were obtained from the National RNAi Core Facility in Taiwan. The target sequences for *ADAM9* were sh*ADAM9*-C (5′-GCCAGAATAACAAAGCCTATT-3′) and sh*ADAM9*-E (5′-CCCAGAGAAGTTCCTATATAT-3′). Lentivirus was packaged in HEK293T cells following the guidelines of the National RNAi Core Facility (http://rnai.genmed.sinica.edu.tw/protocols), and the culture supernatants containing the lentivirus were collected at 48 and 72 h post-transfection. BM7 cells were infected with the lentiviruses overnight in the presence of 8 μg/ml polybrene (Sigma) and were cultured in fresh medium for an additional 24 h. The infected cells were then selected in medium containing 0.4 μg/ml puromycin until the uninfected cells were completely dead.

### Luciferase reporter assay

HEK293 cells were co-transfected with 300 ng of miRNA, 100 ng of the reporter vector containing the *CDH2* 3′-UTR or the mutant *CDH2* 3′-UTR, and 25 ng of the *Renilla* luciferase vector as an internal control. After 48 h, the cells were collected, and the luciferase activities were measured using the Dual-Luciferase Reporter Assay System (Promega, Madison, WI, USA).

### Western blot

The cells were washed twice with phosphate-buffered saline (PBS) (GIBCO, Carlsbad, CA, USA) and lysed in RIPA lysis buffer (Sigma, St. Louis, MO, USA). Protein concentrations were determined using the Protein Assay Reagent (Bio Rad Laboratories, Hercules, CA, USA). Protein samples (30 μg) were loaded on 8% sodium dodecyl sulfate (SDS)-polyacrylamide gels. After electrophoresis, the proteins were transferred to polyvinylidene difluoride (PVDF) membranes (Bio Rad Laboratories, Inc.). Blocking was performed with 5% nonfat milk in a 1X mixture of Tris-buffered saline and Tween 20 (TBST). The Membranes were incubated overnight at 4°C with the following antibodies (at 1∶1000 dilutions in TBST with 5% non-fat milk): ADAM9 (#2099, Cell Signaling, Danvers, MA), CDH1 (E-cadherin, #610404, BD Science, Clontech, Palo Alto, CA, USA), CDH2 (N-Cadherin, #610921, BD Science, Clontech, Palo Alto, CA, USA), VIM (Vimentin, ab8978, Abcam, Cambridge, MA), ACTB (β-actin, ab8226, Abcam, Cambridge, MA) and EF1A (EF1α, #05-235, Millipore, Billerica, MA, USA). After washing and incubation with secondary antibodies (at 1∶2500 dilutions in TBST with 5% non-fat milk) for one h at room temperature, blotted proteins were detected using an enhanced chemiluminescence (ECL) system (Millipore, Billerica, MA, USA) with the BioSpectrum Imaging System (UVP, Upland, CA, USA).

### Cell migration assay

Migration assays were performed using 24-well transwell migration chambers (Corning, Corning, New York, USA) with polyethylene membranes (8 μm pore size). The upper chambers were seeded with 5×10^4^ cells/well in 200 μl of serum-free DF12 or RPMI medium, and the lower chambers were filled with 600 μl of complete medium, which was used as a chemoattractant. The cells were allowed to migrate for 24 h at 37°C. Following incubation, the medium in the upper and lower chambers was removed by aspiration. A methanol-acetic acid (1∶3) mixture was added into the lower chamber to fix the cells. After incubation at room temperature for 20 min, the inserts were washed twice with ddH_2_O. After the well was dried, 0.1% crystal violet (upper: 150 μl; lower: 650 μl) was added, and the inserts were incubated for 20 min at room temperature. After two washes with ddH_2_O, 200 μl of destaining solution was added into the lower chamber of each well to destain the membrane, and the wells were read at an emission wavelength of 570 nm.

For the time-lapse migration assay, BM7 cells with stable, tetracycline-inducible miR-218 expression were cultured on 6-cm dishes coated with collagen (10 μg/ml, 3 ml) and were treated with 20 μg/ml tetracycline for four days. After tetracycline induction, cell movements were monitored using inverted microscopes (Axio Observer Z1, Zeiss, Jena, Germany) with CCD video cameras (AxioCam MRm, Zeiss) at 20 min intervals for a total of 16 h in a 37°C chamber. The accumulated distance was determined by tracking the positions of cell nuclei using the Track Point function of ImageJ.

## Results

### ADAM9 activated CDH2 in aggressive lung adenocarcinoma cells

**Figure 1:**
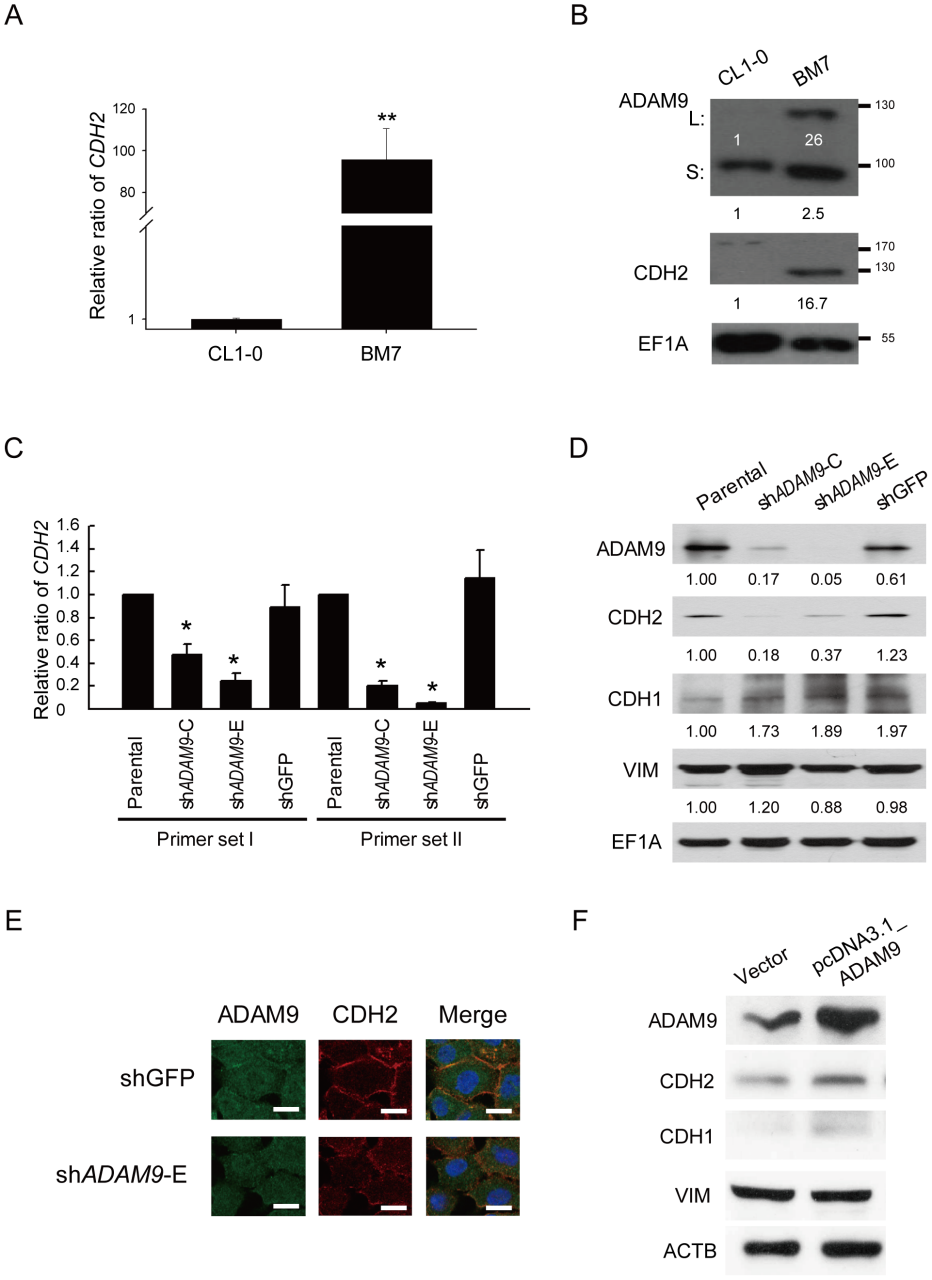
*ADAM9* can activate the expression of *CDH2* in aggressive lung adenocarcinoma cell lines. (A) Quantitative RT-PCR of *CDH2* in the aggressive cell line BM7 and its control line, CL1-0; 18S rRNA was used as a loading control. **, *P*<0.005. (B) Western blot analysis of ADAM9 and CDH2 in BM7 and CL1-0 cells. L: long form of ADAM9; S: short form of ADAM9. EF1A was used as a loading control. EF1A: elongation factor 1 alpha. (C) Relative expression levels of CDH2 in BM7 cells transfected with two siRNAs against ADAM9. Two primer sets (I and II) targeting different *CDH2* regions were used to amplify the *CDH2* products. Two short hairpin RNAs targeted against *ADAM9* (sh*ADAM9*-C & sh*ADAM9*-E) were examined. *HPRT* was used as a loading control. *, *P*<0.05. (D) Western blot analysis of CDH2 in the *ADAM9*-depleted BM7 cells. EF1A was used as a loading control. CDH1: E-cadherin; VIM: vimentin. (E) Immunohistochemistry analysis of ADAM9 and CDH2 in the *ADAM9*-depleted cells. Scale bar: 20 μm. (F) Western blot analysis of CDH2 in parental cells over-expressing *ADAM9*. ACTB was used as a loading control.

To understand whether the expression of *ADAM9* and *CDH2* were correlated with the malignancy of lung adenocarcinoma, we detected the endogenous expression levels of *ADAM9* and *CDH2* using real-time PCR and western blot analyses. Brain-metastatic cell line BM7 and H1299 cells [30] are more aggressive cell lines with high migration ability, whereas the CL1-0 and A549 cell lines were used as controls. As shown in Fig. 1A and 1B, the RNA and protein levels of *CDH2* in the BM7 cells were up-regulated compared with their levels in CL1-0 cells. Similarly, the RNA and protein levels of *CDH2* were more abundant in another lung adenocarcinoma cell line, H1299, compared with A549 cells (Fig. S1 A & B). The amounts of both the long and short forms of ADAM9 were also increased in the aggressive cell lines, including BM7 (Fig. 1B) and H1299 (Fig. S1B).

Next, we assessed whether the expression of *CDH2* changed when the levels of *ADAM9* were altered. First, we used shRNA to knock down *ADAM9* in BM7 cells, and two primer sets were used to measure the expression of *CDH2*. As shown in Fig. 1C, the expression of *CDH2* was significantly down-regulated by both sh*ADAM9* constructs (sh*ADAM9*-C & sh*ADAM9*-E). The protein levels of ADAM9 and CDH2 also decreased when *ADAM9* was knocked down, according to western blot (Fig. 1D) and immunohistochemistry (Fig. 1E) analyses. The amount of CDH1 (E-cadherin) and VIM (vimentin) protein did not change (Fig. 1D). Furthermore, to confirm the relationship between *CDH2* and *ADAM9*, the expression of *CDH2* was measured in BM7 that over-expressed ADAM9. The amount of CDH2 increased in the ADAM9-expressing cells (Fig. 1F). These results indicated that ADAM9 is able to activate CDH2 in aggressive lung adenocarcinoma cells.

### Identification of the differentially expressed miRNAs in aggressive lung adenocarcinoma cells

**Figure 2:**
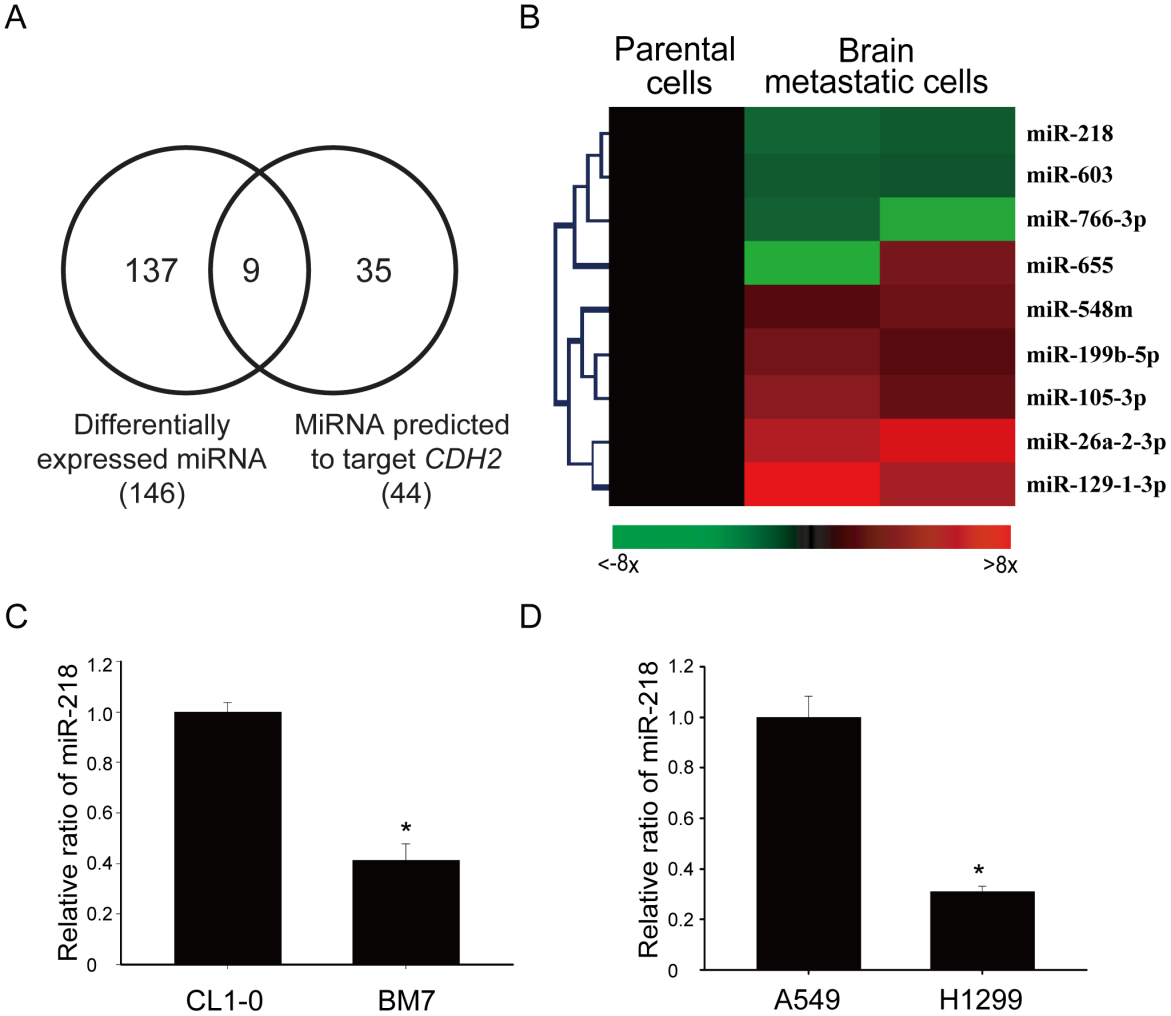
Identification of miRNAs that were differentially expressed in brain metastatic cells and were predicted to target *CDH2*. (A) Venn diagram of miRNAs that were differentially expressed and were predicted to target *CDH2*. (B) Heatmap of the differentially expressed miRNAs that target *CDH2*. Red: up-regulated in brain metastatic cells; green: down-regulated. (C & D) Real-time PCR validation of miR-218 in the brain metastatic cell lines BM7 (C) and H1299 (D). MiR-191 was used as an internal control. *, *P*<0.05.

To investigate which miRNAs could regulate *CDH2* expression in brain metastatic lung cancer cells, we examined the miRNA expression profiles in these cells and their parental cells using an Illumina miRNA microarray. The endogenous expression levels of all miRNAs were examined, and 146 miRNAs were determined to have a >2-fold change in expression in the brain metastatic lung cancer cells. Furthermore, we used several algorithms in the miRSystem program [29] to predict which miRNA targeted *CDH2*. The program uncovered 44 miRNAs that were predicted to target *CDH2*. In total, we identified nine miRNAs that both targeted *CDH2* and showed significant expression changes between the highly metastatic cells and their parental cells (Fig. 2A). Of these miRNAs, four were down-regulated and five were up-regulated in the brain metastatic lung cancer cells (Fig. 2B).

Because *CDH2* was up-regulated in the BM7 cells and miRNAs down-regulate their target genes, we focused on the miRNAs that were down-regulated in the BM7 cells. Of these down-regulated miRNAs, six computational algorithms [29], including DIANA, miRanda, miRBridge, PicTar, rna22, and TargetScan, predicted that miR-218 was the most likely to target *CDH2*. Therefore, we focused on miR-218 for further experiments. We first compared the endogenous expression levels of miR-218 in several lung cancer cell lines (Fig. S2). The results of quantitative RT-PCR validated the down-regulation of miR-218 in the aggressive lung cancer cells, including the BM7 (Fig. 2C) and H1299 (2D) cell lines, compared with their control lines, CL1-0 and A549.

### MiR-218 was generated from pri-mir-218-1 in aggressive lung adenocarcinoma cells

**Figure 3:**
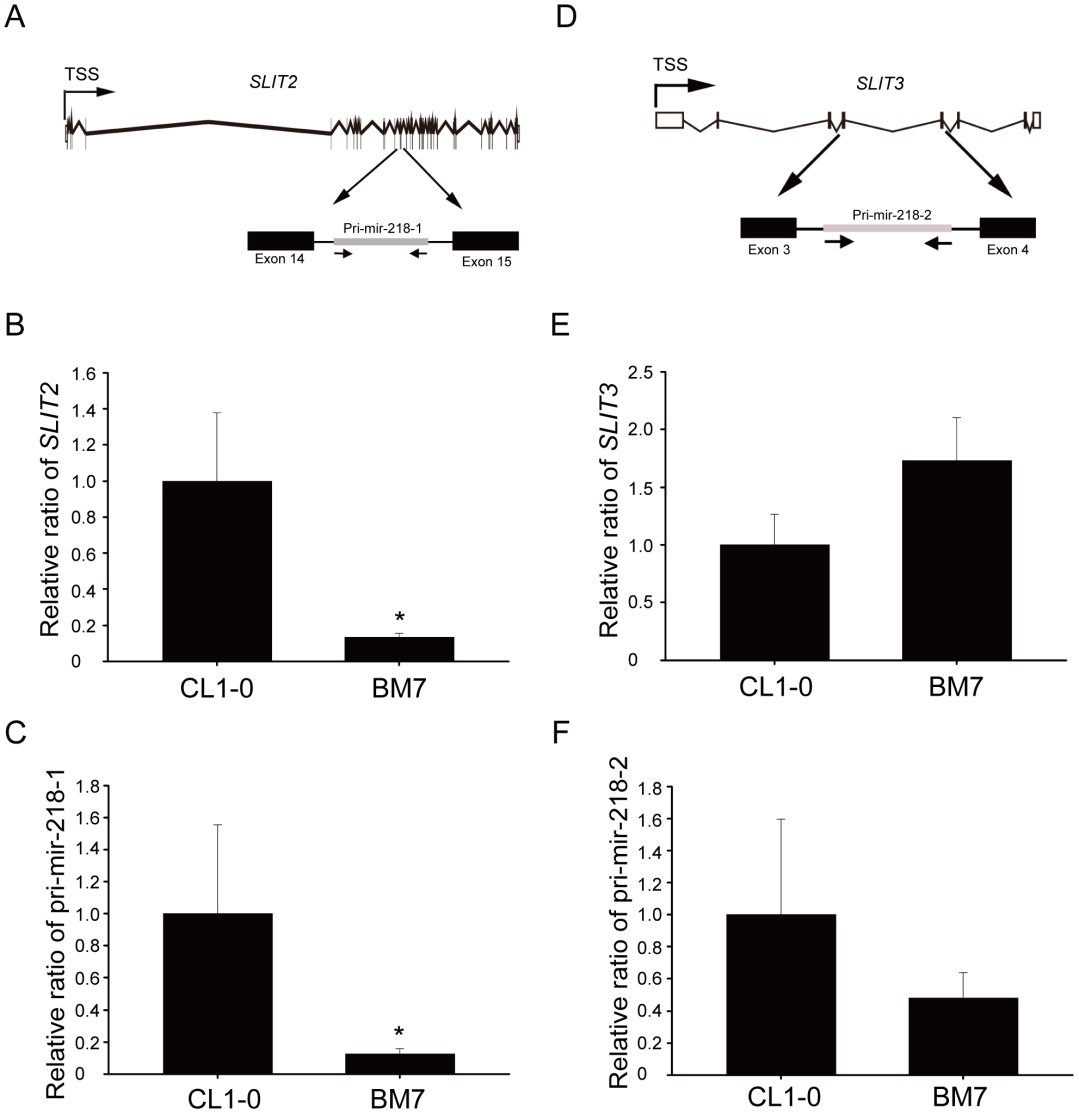
MiR-218 was generated from pri-mir-218-1, which is located in *SLIT2*. (A) Schematic representation of pri-mir-218-1, which is located in the 14^th^ intron of *SLIT2*. TSS: transcription start site. Endogenous expression levels of *SLIT2* (B) and pri-mir-218-1 (C) in BM7 cells. *, *P*<0.05. (D) Schematic representation of pri-mir-218-2, which is located in the 4^th^ intron of *SLIT3*. Expression levels of *SLIT3* (E) and pri-mir-218-2 (F) in BM7 cells.

To investigate which miR-218 precursor was responsible for the down-regulation of miR-218 in the BM7 cells, we examined the expression levels of the miR-218 precursors. The miR-218 precursors were transcribed from the intron of *SLIT2* (pri-mir-218-1) and/or *SLIT3* (pri-mir-218-2) [31]. Pri-mir-218-1 is located within intron 14 of *SLIT2* (Fig. 3A), whereas pri-mir-218-2 is located within intron 4 of *SLIT3* (Fig. 3D). Because intronic miRNAs are co-transcribed with their host genes, the expression levels of *SLIT2*, *SLIT3*, and the mir-218 precursors were measured using real-time PCR. As shown in Fig. 3B and 3C, *SLIT2* and pri-mir-218-1 expression was down-regulated in the BM7 cells. However, *SLIT3* and pri-mir-218-2 expression did not differ between the CL1-0 and BM7 cells (Fig. 3E & 3F). These results indicate that the inhibition of miR-218 in the aggressive BM7 cells was attributed to the suppression of pri-mir-218-1 (*SLIT2*) but not pri-mir-218-2 (*SLIT3*).

### MiR-218 directly regulated *CDH2* in aggressive lung adenocarcinoma cells

**Figure 4:**
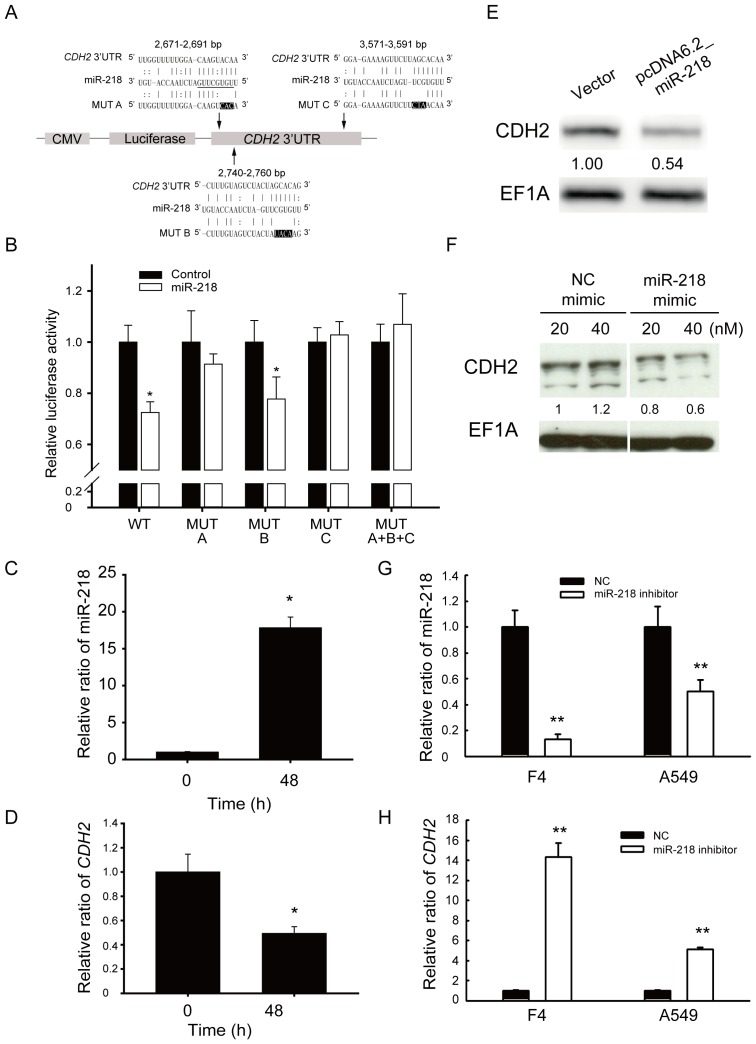
MiR-218 directly regulated *CDH2* activity. (A) Schematic representation of miR-218 targeting the *CDH2* 3′-UTR. Firefly luciferase constructs contained the CMV promoter, luciferase coding region, and a fragment of the *CDH2* 3′-UTR. The locations of the potential miR-218 binding sites are 2,671–2,691 bp, 2,740–2,760 bp, and 3,571–3,591 bp from the transcription start site of *CDH2*. (B) Luciferase assays of miR-218 binding to the *CDH2* 3′-UTR. HEK 293 cells were co-transfected with miR-218, the firefly luciferase construct and the *Renilla* luciferase control for the dual-luciferase assay. The relative luciferase activity represents the dual luciferase activity ratio (firefly/*Renilla* luciferase). WT: wild type; MUT A, B, C: mutation at site A, B, or C, respectively; MUT A+B+C: mutation at sites A, B, and C. *, *P*<0.05. (C) Relative expression levels of miR-218 in BM7 cells over-expressing pri-mir-218. The expression levels of miR-218 were detected using real-time PCR analysis at 0 and 48 h after transfection. MiR-191 was used as an internal control. (D) Relative expression levels of *CDH2* in BM7 cells over-expressing pri-mir-218; 18S rRNA was used as a loading control. (E) Western blot analysis of CDH2 in BM7 cells over-expressing pri-mir-218. EF1A was used as an internal control. (F) Western blot analysis of CDH2 in BM7 cells treated with miR-218 mimic oligonucleotides. (G) Relative expression levels of miR-218 in lung cancer F4 and A549 cells transfected with negative control (NC) or miR-218 inhibitors (200 nM). The expression levels of miR-218 were detected using real-time PCR analysis at 48 h after transfection. U6B was used as an internal control. (H) Relative expression levels of *CDH2* in lung cancer F4 and A549 transfected with NC or miR-218 inhibitors (200 nM); *HPRT* was used as internal control. **, *P*<0.01.

To identify whether miR-218 can bind and regulate *CDH2*, we first used computational algorithms to predict the potential binding sites in the *CDH2* 3′-UTR and examined their interaction using luciferase assays. The locations of the potential binding sites were 2,671–2,691 bp, 2,740–2,760 bp, and 3,571–3,591 bp relative to the transcription start site of *CDH2* (Fig. 4A). Because the seed region of the miRNA, which includes 2 to 8 nucleotides at the 5′-end of the miRNA [21], must be complementary to the 3'-UTR of the target genes, we mutated these binding sites to evaluate which binding sites played important roles (Fig. 4A). By co-transfecting the miR-218 plasmids and the reporter construct, which contained the *CDH2* 3′-UTR behind the luciferase gene (Fig. 4A), we showed that miR-218 was better able to inhibit the luciferase activity compared with the miR-empty vector control (Fig. 4B). When we mutated all the binding sites, the luciferase activity was recovered. Mutation of site A or site C alone, but not site B alone, could relieve the suppression of luciferase activity (Fig. 4B). This result suggested that site B was not a binding site for miR-218. Therefore, we showed that miR-218 can bind to the 3′-UTR of *CDH2* at two binding sites.

To further confirm that *CDH2* could be inhibited by miR-218, we over-expressed miR-218 in metastatic BM7 cells. Real-time PCR showed that miR-218 was significantly up-regulated at 48 h after transfection (Fig. 4C), and the relative mRNA levels of *CDH2* were decreased 0.6-fold in the BM7 cells (Fig. 4D). Western blot analysis also showed that the protein levels of CDH2 were decreased (0.54-fold) following over-expression of miR-218 in BM7 cells (Fig. 4E). Similarly, administration of miR-218 mimic oligonucleotides in BM7 cells resulted in decreased CDH2 expression (Fig. 4F). Furthermore, we over-expressed miR-218 in another lung adenocarcinoma cell line, H1299 (Fig. S3A), and we found that CDH2 was also down-regulated both at the RNA and protein levels (Fig. S3B & C). To confirm this regulation, we further used miR-218 inhibitors to block the levels of miR-218 in lung cancer cells F4 and A549. Real-time PCR showed that miR-218 was significantly decreased at 48 h after transfection (Fig. 4G), and the relative mRNA levels of *CDH2* were increased in these cells (Fig. 4H). These results indicate that miR-218 can down-regulate *CDH2* in aggressive lung adenocarcinoma cells.

### MiR-218 inhibited the migration ability of aggressive lung adenocarcinoma cells

**Figure 5:**
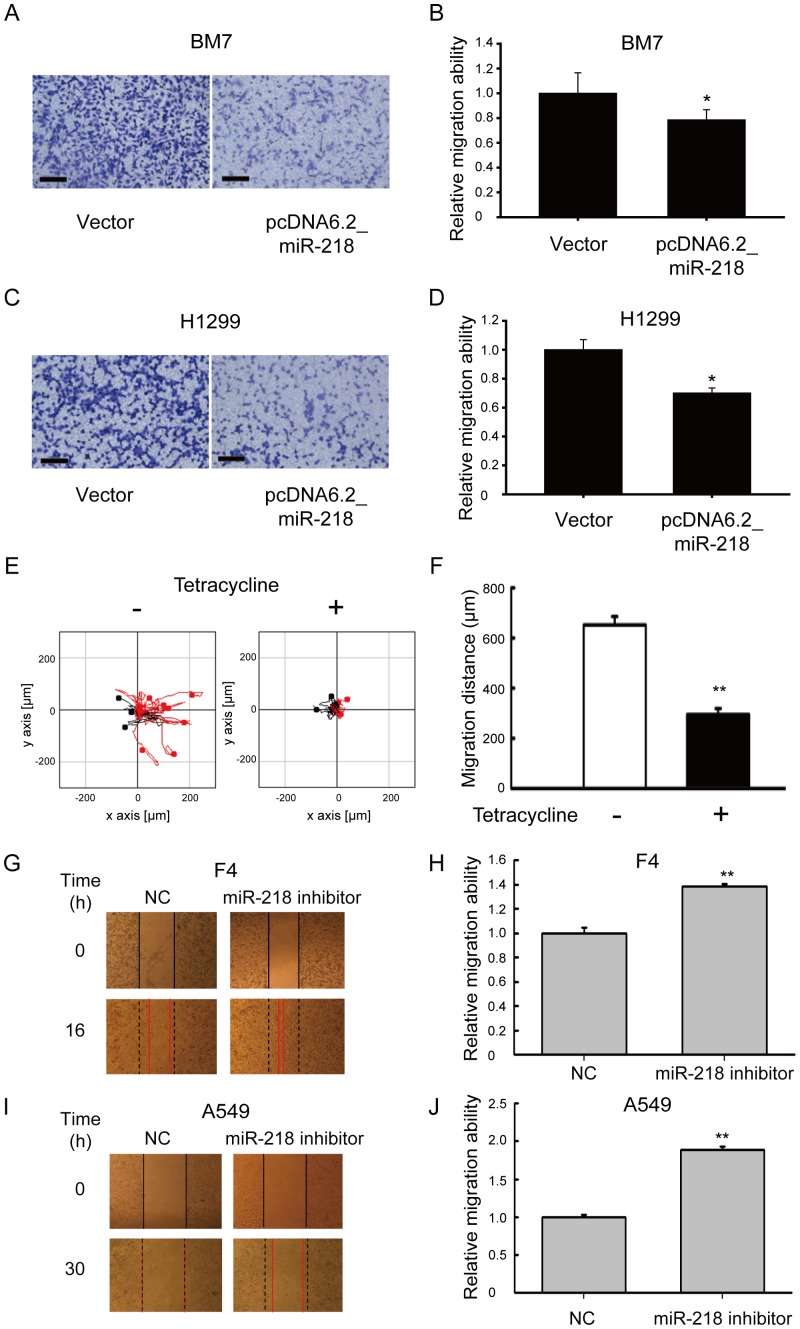
Over-expression of miR-218 suppressed tumor cell mobility. (A) Transwell assays of BM7 cells over-expressing miR-218. Scale bar: 100 μm. (B) Quantitative graph of BM7 cells analyzed in three independent experiments. *, *P*<0.05. (C) Transwell assays of H1299 cells over-expressing miR-218. Scale bar: 100 μm. (D) Quantitative graph of H1299 cells analyzed in three independent experiments. (E) Mobility of BM7 cells over-expressing miR-218. Cell mobility was measured using time-lapse video microscopy in BM7 cells treated with tetracycline for four days. (F) Quantitative graph of the migration distance of the BM7 cells. **, *P*<0.01. (G) Wound healing assays of F4 cells transfected with miR-218 inhibitors. NC or miR-218 inhibitors were transiently transfected into F4 cells for 24 h and then images of wound at 0 and 16 h after wounding were shown. (H) Quantitative graph of wound healing assay in F4 cells transfected with miR-218 inhibitors. Relative migration ability was calculated from four independent experiments. **, *P*<0.01. (I) Wound healing assays of A549 cells transfected with miR-218 inhibitors. NC or miR-218 inhibitors were transiently transfected into A549 cells for 24 h and then images of wound at 0 and 30 h after wounding were shown. (J) Quantitative graph of wound healing assay in A549 cells transfected with miR-218 inhibitors. Relative migration ability was calculated from four independent experiments. **, *P*<0.01.

Previous reports showed that *CDH2* was up-regulated in metastatic cells and induced cell migration [12]. Therefore, we evaluated whether miR-218 could suppress cell migration by targeting *CDH2*. After transfection of miR-218 in both BM7 and H1299 cells, we measured cell migration using transwell migration assays. As shown in Fig. 5A and 5C, the number of migrated cells in the group over-expressing miR-218 was decreased. We quantitated the cell migration ability by detecting the dye used to stain the migrated cells. As shown in Fig. 5B and 5D, the relative cell migration was decreased 0.2-fold in the BM7 cells and 0.3-fold in the H1299 cells. Furthermore, using a tet-on construct to over-express miR-218 in the presence of tetracycline (Fig. S4A & B), we also observed that cell mobility was significantly (*P*<0.01) decreased in the BM7 cells over-expressing miR-218 (Fig. 5E & F). In contrast, blocking miR-218 expression with miR-218 inhibitors in F4 and A549 cells, the migration ability was significantly enhanced in miR-218 inhibitor group compared to negative control (NC) group in F4 (Fig. 5G & H) and A549 cells (Fig. 5I & J). These results indicate that miR-218 can inhibit cell migration by repressing the expression of *CDH2*.

### Relationship between *ADAM9*, miR-218, and *CDH2* in aggressive lung adenocarcinoma cells

**Figure 6:**
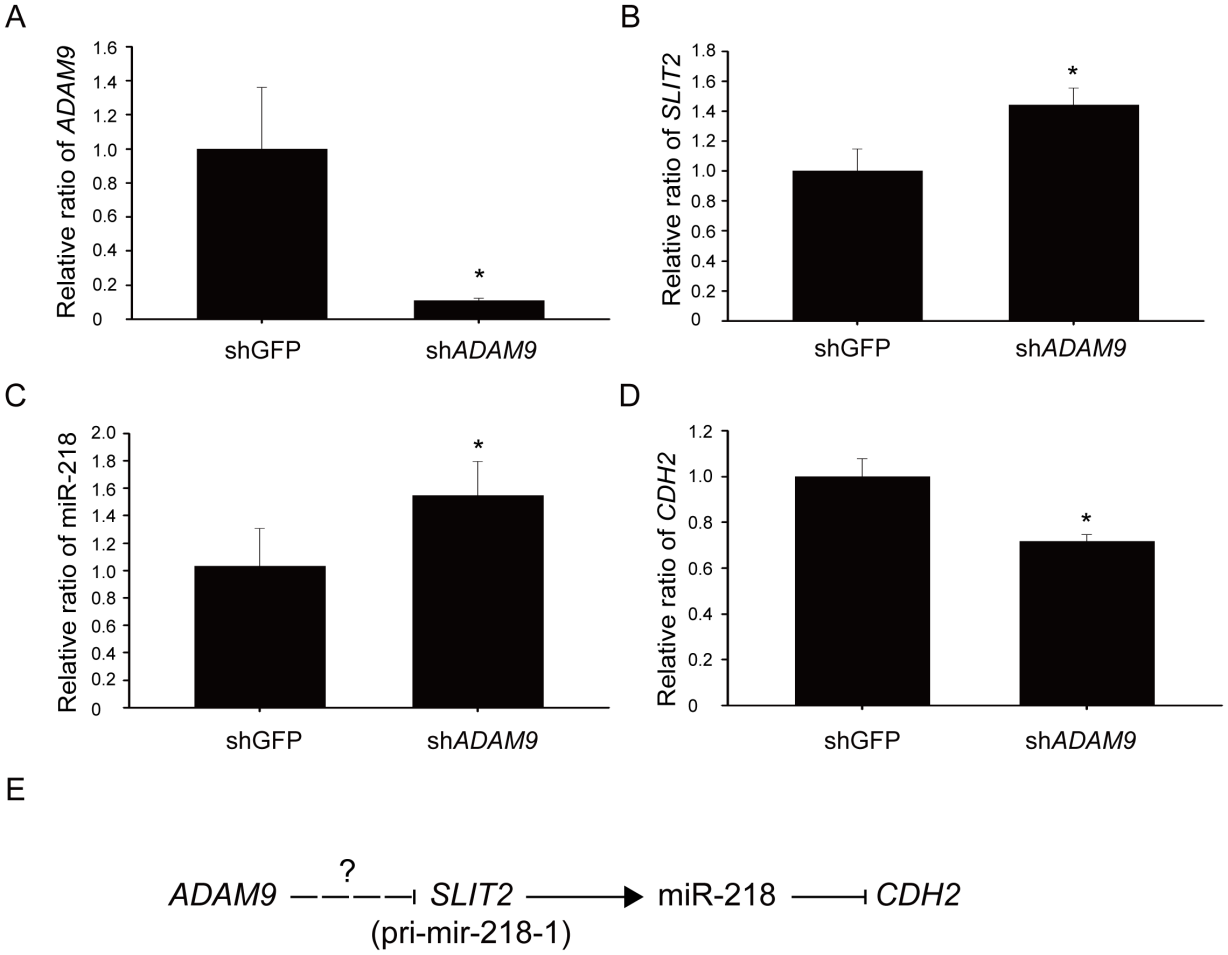
Expression levels of *ADAM9*, *SLIT2*, miR-218, and *CDH2* in *ADAM9*-depleted cells. The expression levels of *ADAM9* (A), *SLIT2* (B), miR-218 (C), and *CDH2* (D) were measured in BM7 cells transfected with short hairpin RNAs targeted against *ADAM9*. 18S rRNA was used as a loading control for *ADAM9*, *SLIT2*, and *CDH2*; miR-191 was used as a loading control for miR-218. *, *P*<0.05. (E) Proposed model for the role of *ADAM9* in the regulation of *CDH2* through the inhibition of miR-218.

Next, to determine the relationship between *ADAM9*, *SLIT2*, miR-218, and *CDH2*, the expression levels of *ADAM9*, *SLIT2*, miR-218, and *CDH2* were measured using real-time PCR in control and ADAM9-knockdown cells. As shown in Fig. 6, *ADAM9* was successfully down-regulated in the BM7-sh*ADAM9* cells (Fig. 6A). Down-regulation of *ADAM9* resulted in the up-regulation of *SLIT2* (Fig. 6B) and miR-218 (Fig. 6C). The up-regulation of miR-218, in turn, reduced the expression of its target gene, *CDH2* (Fig. 6D). Based on these results, we proposed a working model for an ADAM9-miR-218-CDH2 signaling pathway in aggressive lung adenocarcinoma cells (Fig. 6E).

## Discussion

In this study, we demonstrated that endogenous *ADAM9* expression was significantly up-regulated in aggressive lung adenocarcinoma cells, and *ADAM9* could activate the expression of *CDH2*. Down-regulation of miR-218, which resulted from low transcription of pri-mir-218-1, led to CDH2 over-expression in aggressive lung cancer cells. Thus, over-expression of miR-218 could inhibit CDH2 expression and tumor cell mobility. Here, we illustrate the mechanism by which *ADAM9* activates *CDH2*, which may be due to the release of miR-218 inhibition of *CDH2*.

Previously, miR-218 was mostly regarded as a tumor suppressor in many cancers. For example, miR-218 could inhibit migration, invasion, and proliferation of glioma cells [32], head and neck squamous cell carcinoma cells [33], cervical squamous cell carcinoma cells, nasopharyngeal cancer cells [34], and gastric cancer cells [35]. MicroRNA-218 could also inhibit cell cycle progression, promote apoptosis in colon cancer [36], and increase the chemosensitivity of cervical cancer cells to cisplatin [37]. In primary non-small cell lung cancer, miR-218 was deleted or down-regulated, and its expression could be used to predict survival and relapse [38]. When miR-218 expression was low in lung cancer patients, their clinical outcomes were poor [38]. Our findings were consistent with these previous results, thus confirming the tumor suppressor role of miR-218. In contrast, only one study reported that miR-218 was a potent activator of Wnt signaling, contributed to osteoblastogenesis, and facilitated the metastasis of breast cancer cells into the bone [39].

Several targets of miR-218 have been reported, including *BMI1* [36], *PXN* [38], *BIRC5* [34], *GJA1* [34], laminin-332 [33], and *ROBO1* [34], [35]. In particular, the miRNA-218 and ROBO1 signaling axis has been studied extensively and correlates with metastasis and vascular patterning in pancreatic and nasopharyngeal cancers [40], [41]. In this study, we demonstrated that miR-218 can directly bind to the 3′-UTR of *CDH2* at two binding sites (2,671–2,691 bp and 3,571–3,591 bp) using luciferase reporter assays. Interestingly, the binding site at 3,571–3,591 bp has also been reported in bovine cells [42], which supports our finding that miR-218 targets *CDH2*. Furthermore, over-expressing miR-218 by transfection of an expression vector or miR-218 mimic oligonucleotides resulted in a dramatic decrease in the CDH2 protein level, indicating that *CDH2* was indeed a target gene of miR-218.

In this study, we observed low expression levels of miR-218 in the aggressive lung cancer cell lines BM7 and H1299 (Fig. 2A & 2B). We further explored this down-regulation by examining the expression of the precursor miRNAs of miR-218. The miR-218 transcripts are located within the introns of *SLIT2* (pri-mir-218-1) and *SLIT3* (pri-mir-218-2), which were reported to function as tumor suppressors [43]. The expression levels of *SLIT2*, *SLIT3*, pri-mir-218-1, and pri-mir-218-2 were detected using real-time PCR. We found that the down-regulation of miR-218 in lung adenocarcinoma cells was related to the expression of *SLIT2*. Hyper-methylation of the CpG-islands in *SLIT2* [44] and copy number losses of *SLIT2* have been reported [45]. Additionally, *SLIT2* could suppress cell migration through the regulation of beta-catenin [46], the AKT-GSK3β signaling pathway [47], and the ROBO1 signaling pathway [34]. However, in gastric cancer and thyroid cancer, it was shown that down-regulation of miR-218 was attributed to low expression levels of *SLIT3* [31], [34], and restoring the expression of miR-218-2 and *SLIT3* could repress cell invasion and migration [31]. The difference between lung cancer and gastric cancer may be due to the tissue specificity of the miRNA precursors that result in mature miR-218.

ADAM9 has two isoforms, including a shorter ADAM9-secreted (ADAM9-S) transcript and a transmembrane protein, ADAM9-long (ADAM9-L). ADAM9 is typically regarded as oncogene in many cancers, such as oral squamous cell carcinomas [48], breast tumors [49], prostate cancer [50], and renal cell cancer [51]. Inhibition of ADAM9 expression can sensitize prostate cancer cells to radiation and chemotherapy [50]. However, the ADAM9 splice variants have opposing effects on breast cancer cell migration [52]. ADAM9-S promoted breast cancer cell migration, whereas ADAM9-L suppressed cell migration. Therefore, a key determinant in the manifestation of aggressive migratory phenotypes is the relative levels of the membrane-tethered and secreted variants of ADAM9. In our results, the relative ratio of the short form to the long form was higher in the BM7 cell line compared with the CL1-0 cell line, which corresponded to the aggressiveness of BM7. Moreover, we found that down-regulation of *ADAM9* could up-regulate *SLIT2*. However, there is no direct evidence indicating that ADAM9 can regulate *SLIT2*; thus, more experiments are needed to explore this relationship.

In conclusion, brain metastasis of lung cancer is one of the main reasons for the high mortality of this disease. MicroRNAs have been reported to modulate tumor metastasis. We demonstrated that down-regulation of miR-218 was attributed to low expression of its host gene, *SLIT2*, and its precursor, pri-mir-218-1. Although there was no direct evidence that *ADAM9* regulates *SLIT2*, the down-regulation of *ADAM9* resulted in the up-regulation of *SLIT2* and miR-218, which in turn down-regulated *CDH2* (Fig. 6). Overall, this study increases our understanding of how lung cancer cells metastasize to the brain and may result in the development of new therapeutic strategies for lung cancer.

## Supporting Information

Figure S1 **ADAM9 and CDH2 were up-regulated in aggressive lung adenocarcinoma cell lines.** (A) Quantitative RT-PCR of *CDH2* in the aggressive cell line H1299 and control A549 cells; 18S rRNA was used as a loading control. **, *P*<0.005. (B) Western blot analysis of ADAM9 and CDH2 in H1299 and A549 cells. L: long form of ADAM9; S: short form of ADAM9. EF1A was used as a loading control.
(TIF)

Figure S2 **Relative expression levels of miR-218 in lung cancer cell lines.** A549, H1299, CL1-0, F4, and BM7 were described in Materials and Methods. Immortalized normal lung epithelial cells (HBEC-3KT) were kindly provided by Dr. John D Minna [53]. PC-9 was a gift from Dr. Mien-Chie Hung [54].
(TIF)

Figure S3 **Over-expression of miR-218 suppressed the expression of** ***CDH2*** **.** (A) Relative expression levels of miR-218 in H1299 cells over-expressing miR-218. The expression levels of miR-218 were detected using real-time PCR at 0 and 48 h after transfection. MiR-191 was used as an internal control. *, *P*<0.05. (B) Relative expression levels of *CDH2* in H1299 cells over-expressing miR-218. 18S rRNA was used as a loading control. *, *P*<0.05. (C) Western blot analysis of CDH2 in H1299 cells over-expressing miR-218. EF1A was used as an internal control.
(TIF)

Figure S4 **Over-expression of miR-218 using a tetracycline-inducible construct.** (A) Relative expression levels of miR-218 in BM7 cells treated with different doses of tetracycline. Tet: tetracycline; Pool: pool population; Clone 2: a selected stable clone. (B) Western blot analysis of CDH2 in tetracycline-induced BM7 cells overexpressing miR-218. EF1A: EF1α.
(TIF)
