# Supplementary figures and images for: ADAM9 Up-Regulates N-Cadherin via miR-218 Suppression in Lung Adenocarcinoma Cells
Source: PLoS One. 2014 Apr 4;9(4):e94065. doi: 10.1371/journal.pone.0094065 (PMC3976390; doi:10.1371/journal.pone.0094065)

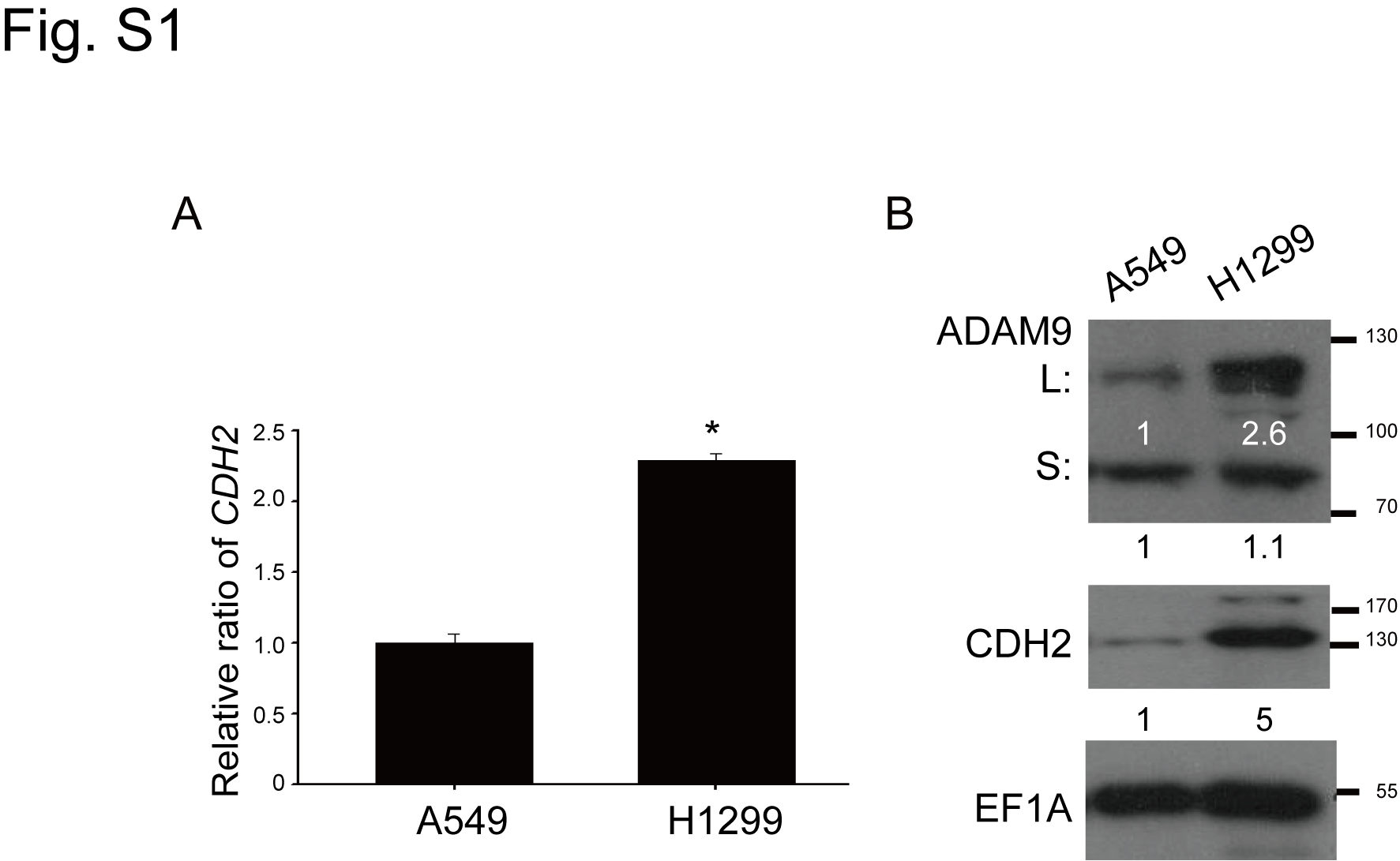

Supplement: Figure S1 — ADAM9 and CDH2 were up-regulated in aggressive lung adenocarcinoma cell lines. (A) Quantitative RT-PCR of CDH2 in the aggressive cell line H1299 and control A549 cells; 18S rRNA was used as a loading control. **, P<0.005. (B) Western blot analysis of ADAM9 and CDH2 in H1299 and A549 cells. L: long form of ADAM9; S: short form of ADAM9. EF1A was used as a loading control. (TIF) [file pone.0094065.s001.tif]

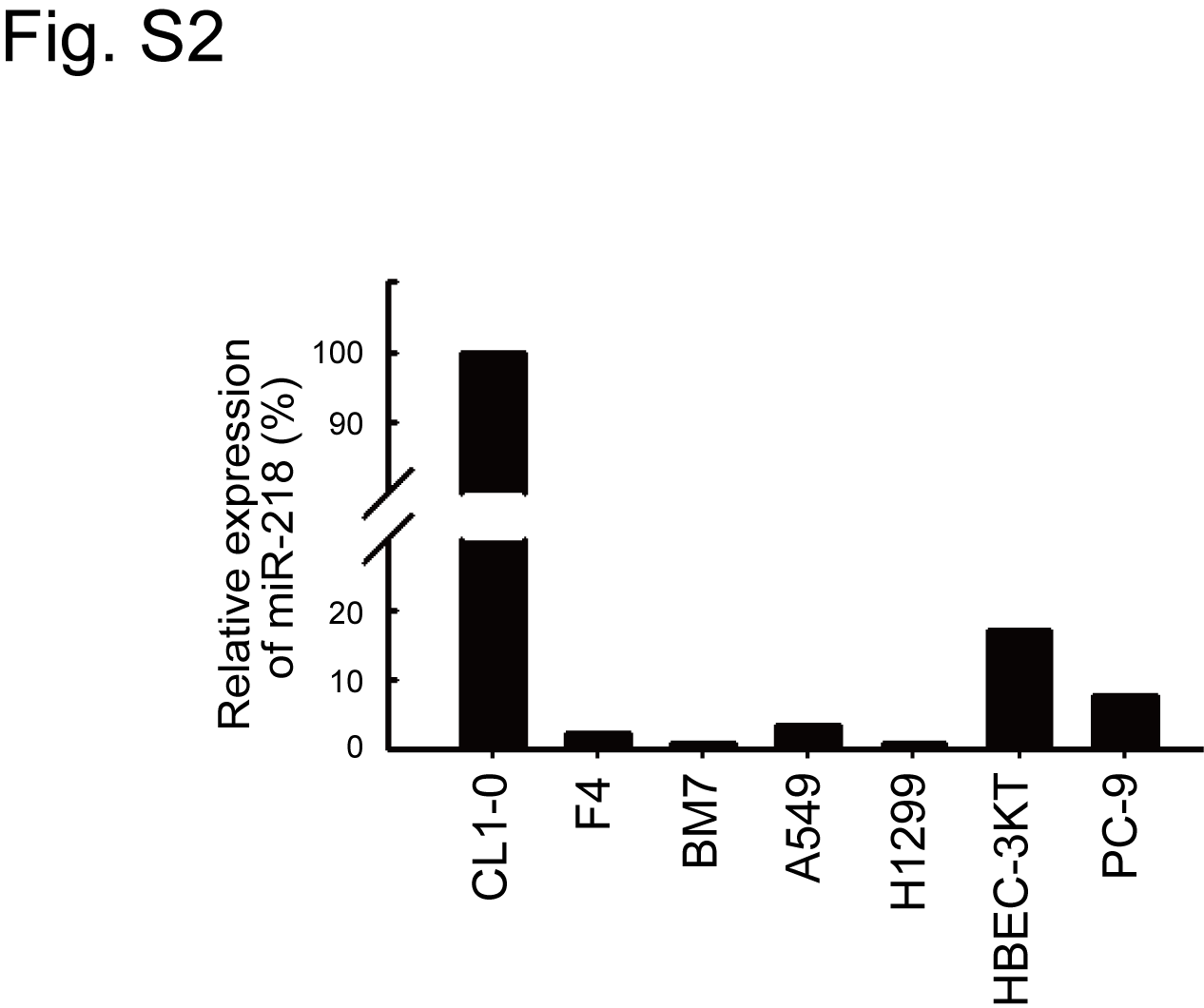

Supplement: Figure S2 — Relative expression levels of miR-218 in lung cancer cell lines. A549, H1299, CL1-0, F4, and BM7 were described in Materials and Methods. Immortalized normal lung epithelial cells (HBEC-3KT) were kindly provided by Dr. John D Minna [53]. PC-9 was a gift from Dr. Mien-Chie Hung [54]. (TIF) [file pone.0094065.s002.tif]

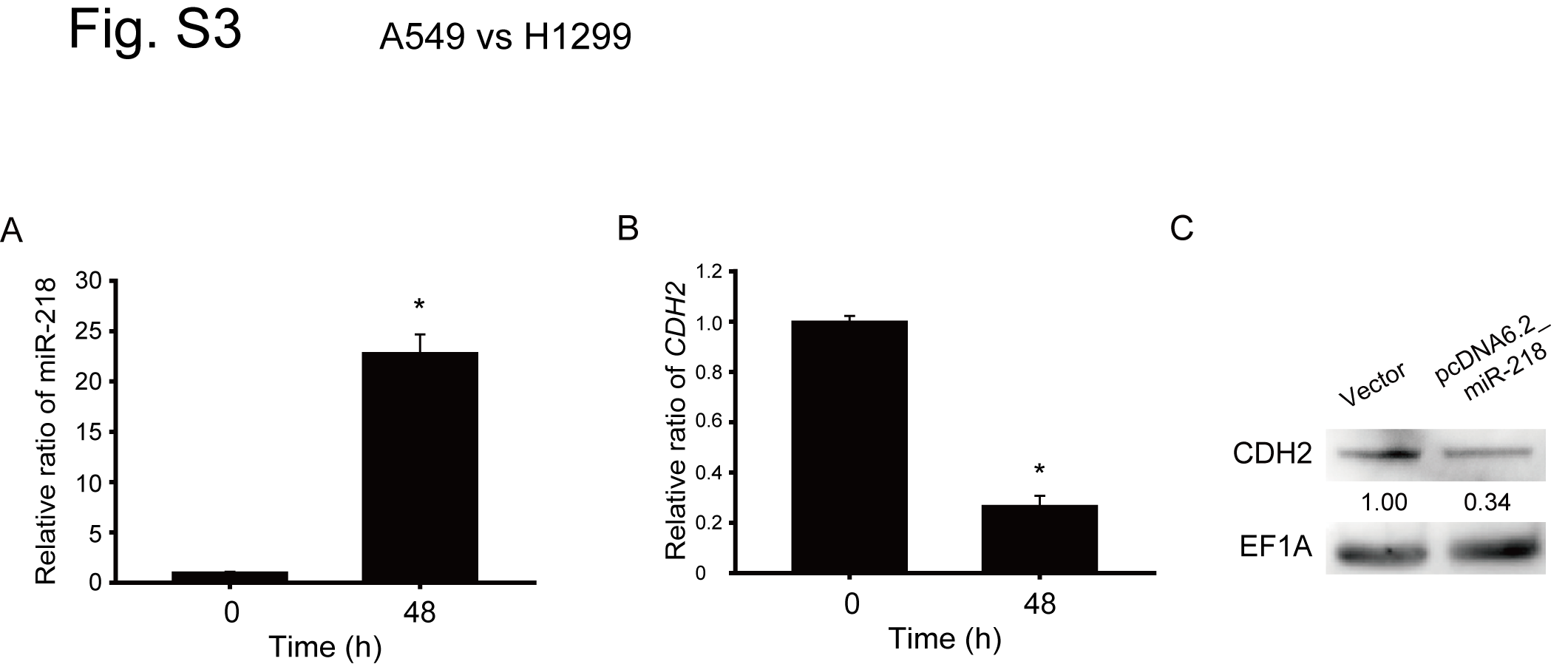

Supplement: Figure S3 — Over-expression of miR-218 suppressed the expression of CDH2 . (A) Relative expression levels of miR-218 in H1299 cells over-expressing miR-218. The expression levels of miR-218 were detected using real-time PCR at 0 and 48 h after transfection. MiR-191 was used as an internal control. *, P<0.05. (B) Relative expression levels of CDH2 in H1299 cells over-expressing miR-218. 18S rRNA was used as a loading control. *, P<0.05. (C) Western blot analysis of CDH2 in H1299 cells over-expressing miR-218. EF1A was used as an internal control. (TIF) [file pone.0094065.s003.tif]

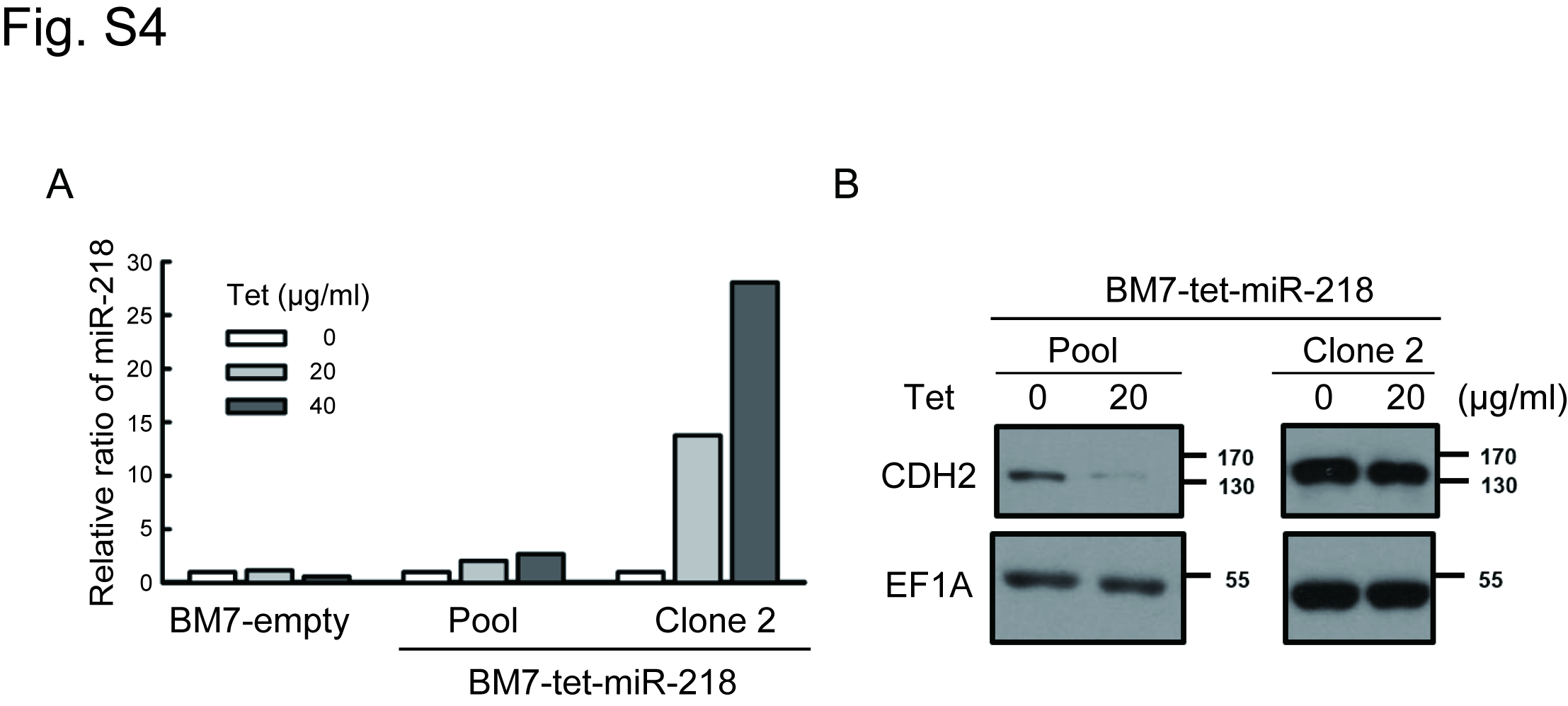

Supplement: Figure S4 — Over-expression of miR-218 using a tetracycline-inducible construct. (A) Relative expression levels of miR-218 in BM7 cells treated with different doses of tetracycline. Tet: tetracycline; Pool: pool population; Clone 2: a selected stable clone. (B) Western blot analysis of CDH2 in tetracycline-induced BM7 cells overexpressing miR-218. EF1A: EF1α. (TIF) [file pone.0094065.s004.tif]
